# Arteriovenous malformations within jejunal diverticulosis: case report and literature review

**DOI:** 10.1186/s12893-019-0538-0

**Published:** 2019-06-27

**Authors:** Tagleb S. Mazahreh, Abdelwahab J. Aleshawi, Mohammed S. Alorjani, Rasheed Elayyan, Nabil A. Al-Zoubi

**Affiliations:** 1 Department of General Surgery and Urology, Faculty of Medicine, Jordan University of Science & Technology, P. O. Box: 3030, Irbid, 22110 Jordan; 2 Department of Pathology and Microbiology, Faculty of Medicine, Jordan University of Science & Technology, Irbid, 22110 Jordan

**Keywords:** Angiodysplasia, Jejunum, Angiography, Diverticulosis

## Abstract

**Background:**

Jejunal diverticula are the rarest of all small bowel diverticula. Most patients with jejunal diverticula are asymptomatic. Major complications include diverticulitis, gastrointestinal hemorrhage, intestinal obstruction and perforation. The hemorrhage has been attributed to diverticulitis with ulceration, diverticulosis associated with trauma and irritation disorder. However, only six cases reported the arteriovenous malformations within jejunal diverticulosis to be the cause of hemorrhage.

**Case presentation:**

We present a case of arteriovenous malformations within jejunal diverticulosis in a 68-year-old male presented with lower gastrointestinal bleeding. After admission and stabilization, upper and lower endoscopies were performed without demonstrating the bleeding site. They only revealed clotted and red blood throughout the colon. Technetium-labeled red blood cell bleeding scan, endoscopic capsule, and selective angiography were performed to localize the site of bleeding without significant findings. As the clinical status of the patient deteriorated, exploratory laparotomy was performed urgently. Extensive jejunal saccular pouches were found 10 cm distal to duodenojejunal junction extending 1.6 m distally. Segmental resection was performed with side to side primary anastomosis. Microscopic examination of the specimen revealed many diverticula. He was followed up 2 years after that without complications.

**Conclusion:**

We report yet the seventh case jejunal diverticulosis with the presence of angiodysplasia, in hope of expanding the knowledge of a rare occurrence and increasing the demand for further research about the etiology, clinical impact and treatment of such anomalies coexistence. This case also highlights the importance of considering the diagnosis of AVMs within jejunal diverticulosis in the presence of uncontrollable blood loss in the pre- or intra- operatively diagnosed jejunal diverticulosis and the urgent need for surgical intervention. In addition, the diagnostic tests should be performed close to the bleeding episode.

## Background

Small intestinal diverticulosis is defined as the presence of multiple sac-like mucosal herniations through weak points in the intestinal wall. Jejunal diverticula are the rarest form of all small bowel diverticula. Small bowel contrast imaging studies reported the incidence of 0.5 to 2.3% and 0.3 to 4.5% in the autopsy studies [1, 2].

Most patients with jejunal diverticula are asymptomatic [3, 4]. However, jejunal diverticulosis may present more acutely as diverticulitis, gastrointestinal bleeding, intestinal obstruction and perforation [3, 5]. Hemorrhage from such diverticula is a well-recognized complication which is considered to be a result of erosion of normal blood vessels [3–5]. However, significant bleeding from coexisting lesions within the diverticula, for example, arteriovenous malformations (AVMs) is very unusual and rare [6]. We present a case of AVM within jejunal diverticulosis in a 68-year-old male presented with lower gastrointestinal bleeding.

## Case presentation

A 68-year-old asthmatic male patient presented to our center with 12 days history of melena. He denied any previous episode of melena or hematochezia or bleeding from another site. The patient did not have any other associated symptom, and had no other co-morbidities, or medication use. Upon referral, he was uncomfortable and looked pale. He was vitally unstable, with a blood pressure of 90/60 mmHg and a pulse rate of 120 beats/min. The examination revealed the presence of clotted blood on the anal verge, and some tarry stool on digital rectal examination. The hemoglobin level was 7.7 g/dl, the hematocrit was 22.8, and the blood urea nitrogen was 8 mg/dl. The prothrombin time and the partial thromboplastin time were normal.

Resuscitation was performed with transfusion of 2 units of packed red blood cells and intravenous fluids. He was admitted to the ICU for intensive monitoring. After admission and stabilization, upper and lower endoscopies were performed without demonstrating the bleeding site. They only revealed clotted and red blood throughout the colon.

Technetium-labeled red blood cell bleeding scan was done to localize the site of bleeding. This scan showed no evidence of early focal increased uptake in the abdomen to indicate active gastrointestinal bleeding during early images, but in the delayed images, it revealed that there was a focal uptake in the right and transverse colon. After that, capsule endoscopy was also performed without findings. As the angiography became available, the patient underwent selective angiography without findings noted at that time. These tests were inconclusive because they were performed while the episodes of bleeding ceased.

**Fig. 1:**
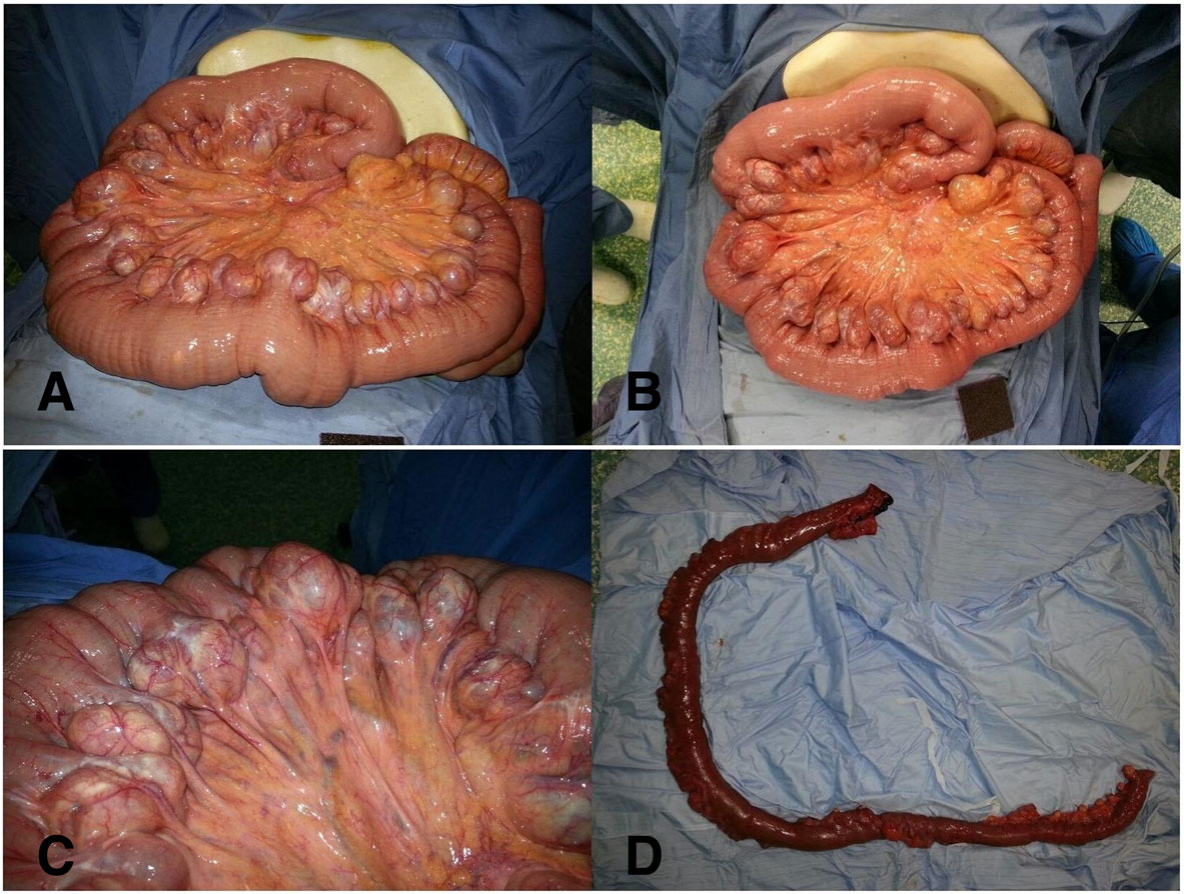
Intraoperative findings. **a** and **b** Jejunal saccular pouches were found 10 cm distal to duodenojejunal junction extending 1.6 m distally. **c** Extensive jejunal saccular pouches. **d** After resection

After 8 days of conservative management and negative investigations to define the cause of the bleeding, a sudden drop in hemoglobin level from 10.8 mg/dl to 6.9 mg/dl occurred over 12 h, which mandated operative management. Exploratory laparotomy was performed. Extensive jejunal saccular pouches were found 10 cm distal to duodenojejunal junction extending 1.6 m distally Fig. 1. The bleeding was difficult to control and the decision to clamp the major branched was performed. Division of the small bowel proximal and distal to the diseased part using gastrointestinal stapler was performed with side to side primary anastomosis Fig. 1. The specimen was a part of small bowel, 117 cm in length, with congested wall and multiple pouches at the mesenteric site. Opening of the specimen showed normally looking mucosa with active bleeding that stopped after awhile. No polyps or masses were detected. We reviewed the angiography achieve after that and a suspicious shadow reflecting the diverticular outpouching was detected.

**Fig. 2:**
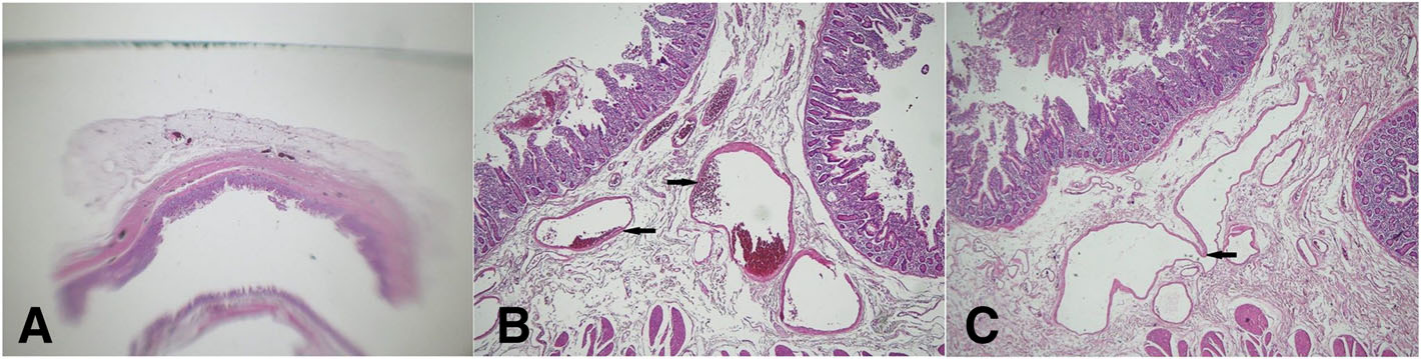
Microscopic examination. **a** It revealed the presence of AVMs within the jejunal diverticula. **b** and **c** There were numerous dilated thick- and thin-walled small blood vessels in the submucosa

Microscopic examination of the specimen revealed many diverticula; some of which being true diverticula, while the others are devoid of muscularis propria (false diverticulae). Within the diverticula and in the intervening portions of the bowel wall, there were numerous dilated thick- and thin-walled small blood vessels in the submucosa. Additionally, submucosal intermediate-sized vascular clusters and feeder vessels in the muscularis propria and serosa were present. The overall features were those of small intestinal diverticulosis and arteriovenous malformations. The latter involves the diverticula and intervening portions of the bowel wall Fig. 2.

Postoperatively, the patient was doing well, discharged home on day 5 postoperatively, with an uneventful postoperative course. He was followed up 2 years after that without complications.

## Discussion and conclusion

To best of our knowledge, only six cases were reported to have AVMs within jejunal diverticulosis [7–12]. Jones et al. reported in 1990 the first case of AVM within jejunal diverticulosis in 68-year-old male [7]. After that, Elsti et al. reported another case of jejunal diverticulosis with the coexistence of AVM in 62-year-old female [8]. In 2017, Alva et al. reported the sixth case in 21-year-old male [12]. Longo and Vernava did not reported any AVM complicated the presence of jejunal diverticulosis in their review article [13]. In addition, Donald did not detect the presence of AVM in his series of complicated cases of jejunal diverticulosis [14]. The clinical data for the six reported cases in addition to the presented case were summarized in Table 1. The mean age for patients was 66.6. Both sex affected equally, and main presentation is melena with circulatory collapse.

**Table 1:** Only 7 cases report the coexistence of AVMs within jejunal diverticulum

Case#	Age	Sex	Presentation	diagnostic Radiological test	Pathological confirmation	diagnostic Endoscopic procedure	Treatment	Author/Year
1	68	Male	IDA	Angiography was diagnostic for AVM	Yes	–	Segmental resection	Jones/1990 [7]
2	62	Female	Melena	Angiography was diagnostic for AVM	–	Not diagnostic	Surgery	Elsti/1998 [8]
3	70	Female	Massive melena	–	Yes	Not diagnostic	Segmental resection	Kawamura/2000 [9]
4	91	Female	Hematochezia and hypotension	Selective angiography identified the bleeder vessel only	Yes	Not diagnostic	Segmental resection	Lee/2009 [10]
5	86	Female	IDA, hematochezia	–	–	Single balloon enteroscopy	Argon plasma coagulation	Fernandes/2015 [11]
6	21	Male	Melena, hyptension	Angiography was diagnostic for AVM	Yes	Not diagnostic	Segmental resection	Alva/2017 [12]
7	68	Male	Melena, hypotesion	Angiography was suspicious for diverticula	Yes	Not diagnostic	Segmental resection	Present case

Small bowel diverticula were first described by Soemmering and Baillie in 1794 and by Sir Astley Cooper in 1807 [1]. Jejunal diverticula are acquired outpouchings and sac-like mucosal herniations detected on the mesenteric border of the jejunum [15]. These lesions are mostly multiple, but the number decreases distally. The mean age of detection is the sixth and seventh decades of life and have slight male predominance [16]. The sizes of these diverticula vary between a few millimeters to greater than ten centimeters [15, 16]. The pathogenesis of these lesions is multifactorial. The site of herniation appears to occur in the points where blood vessels penetrate through the mesentery into the bowel wall. This theory is augmented by the fact that jejunal diverticula most readily in the proximal jejunum and distal ileum, where the vasa recti of greatest diameter lie. This theory explains their common location at the mesenteric side of the bowel [13]. The second theory is the abnormal contractions of the intestinal wall muscle [17]. Also, the obesity, venous stasis, and increased intraluminal pressure due to constipation may have a role in the development of jejunal diverticula [18].

Most patients with jejunoileal diverticular disease are asymptomatic. In small percentage of symptomatic patients, jejunal diverticulosis will present as malabsorption, hemorrhage, inflammation, or obstruction [19]. Braithwaite reported the first case of bleeding from jejunal diverticulosis in 1923 [20]. Jejunal diverticulosis is an unusual and rare cause of massive small bowel hemorrhage; however, it could be a fatal complication [15, 16]. These patients mostly present with hematochezia but can also present with melena and hematemesis [15, 16]. This hemorrhage maybe due to diverticulitis with ulceration, diverticulosis associated with trauma and irritation disorder [13, 15, 16]. More significantly, the hemorrhage could be due to AVMs as the presented case [7–12]. In general, AVMs or angiodysplasia of the gastrointestinal tract is an acquired lesion of small submucosal and mucosal blood vessels which can give rise to hemorrhage [7]. It is commonly found the colon but also occurs less frequently in the small intestine and stomach [7]. The diagnostic modalities for AVMs are mainly endoscopic procedures [21, 22]. However, in cases of massive gastrointestinal hemorrhage or small bowel location, selective angiography or scintigraphy through labeling the red blood cells can detect these lesions. No single diagnostic test has 100% sensitivity, however, the pathological studies remain the definitive diagnostic procedure in case of surgical resection [23–25].

To our knowledge, if the aforementioned imaging or interventional procedures fail to detect AVMs within jejunal diverticula, we should suspect these lesions in two situations. If the selective angiography is able detect the bleeder vessel or the diverticulum while coiling this vessel fails to stop the bleeding. Also, when the resected bowel that contains the diverticula persists to bleed profusely or when the surgeons need to clamp the main mesenteric branches to cease the bleeding intraoperatively. In the presented case, radiolabeled red blood cells had no role in detecting either the diverticula or the AVMs. The selective angiography contributed to the suspicion of the presence jejunal diverticula. In addition, esophagogastroduodenoscopy, colonoscopy and capsule endoscopy did not have any role in detecting the site of bleeding. The best treatment method of jejunal diverticulosis with AVMs is segmental resection with primary anastomosis. Argon plasma coagulation was utilized in one case [12].

We report yet the seventh case jejunal diverticulosis with the presence of angiodysplasia, in hope of expanding the knowledge of a rare occurrence and increasing the demand for further research about the etiology, clinical impact and treatment of such anomalies coexistence. This case also highlights the importance of considering the diagnosis of AVMs within jejunal diverticulosis in the presence of uncontrollable blood loss in the pre- or intra- operatively diagnosed jejunal diverticulosis and the urgent need for surgical intervention. Moreover, the most appropriate tests should be performed as close as possible to the bleeding episode in order to locate and identify the cause of bleeding before surgery.

## Data Availability

Data sharing does not apply to this article as no datasets were generated or analyzed during the current study.

## Abbreviation

AVMs: Arteriovenous malformations
